# Clinical Analysis of 10 AIDS Patients with Malignant Lymphoma

**DOI:** 10.3969/j.issn.2095-3941.2012.02.006

**Published:** 2012-06

**Authors:** Gui-ju Gao, Di Yang, Ke-ke Lin, Jiang Xiao, Xin Li, Hong-yuan Liang, Long Liu, Ning Han, Hong-xin Zhao

**Affiliations:** 1 Beijing Ditan Hospital, Capital Medical University, Beijing 100015, China; 2 School of Nursing, Peking University, Beijing 100191, China

**Keywords:** acquired immunodeficiency syndrome (AIDS), malignant lymphoma, chemotherapy, HAART, prognosis

## Abstract

**Objective:**

This work summarizes the clinical features and treatment of 10 AIDS patients with malignant lymphoma.

**Methods:**

A total of 10 AIDS patients with malignant lymphoma seen in Beijing Ditan Hospital since 2009 were enrolled. Clinical manifestations, pathological examinations, immunity levels, Epstein-Barr virus antibody examinations, complications, treatments, and outcomes were retrospectively analyzed.

**Results:**

The main clinical manifestations of these patients included intermittent fever in 2 cases, neck masses and fever in 3 cases, auxiliary lymph node enlargement in 2 cases, and abdominal pain and bloating with fever in 3 cases. Up to 7 patients were pathologically diagnosed with diffuse large B cell lymphoma (DLBCL), and 3 patients were pathologically diagnosed with Burkitt’s lymphoma. Up to 8 patients had CD4 cell counts below 200/µL, and 2 patients had a level of more than 200/µL. Up to 7 patients were negative for EBV-IgM antibodies and 3 patients were not examined. Six patients underwent different chemotherapy and their prognoses were different. One patient with Burkitt’s lymphoma alternatively took CODOXM and IVAC for 3 turns after VP chemotherapy; 1 patient with liver metastasis took R-CHOP 5 times, then changed therapy regimen to R-MINE and MINE. One patient with adrenal DLBCL took CHOP 6 times. Three patients with DLBCL took CHOP 1 or 2 times. Four patients gave up treatment. Various infections and side effects occurred, including bone marrow suppression, gastrointestinal bleeding, and renal dysfunction during chemotherapy. Six patients took HAART, and 4 did not. Six patients died, whereas 3 patients got improved; and 1 patient was discharged.

**Conclusions:**

AIDS patients with malignant lymphoma had various clinical manifestations, were immunocompromised, and had multiple metastases when they were admitted; they were already in the interim or late stage of lymphoma. Chemotherapy was not effective, and additional complications occurred. HAART failed to improve patient prognosis, and the overall prognosis was poor.

## Introduction

Acquired Immunodeficiency Syndrome (AIDS) patients often develop opportunistic infections and malignant tumors because of their low immune function. Studies found that 3.3% of newly diagnosed AIDS patients had non-Hodgkin’s lymphoma (NHL) ^[^^1^^]^. Most of these patients were in the middle or late stages of the disease when they saw a doctor. Thus, monotherapy is often ineffective ^[^^2^^]^. The most effective method is combined treatment. This study summarizes and analyzes the clinical data and treatment of 10 AIDS patients with malignant lymphoma.

## Patients and Methods

### Patients

The First Department of Infectious Diseases of the Beijing Ditan Hospital admitted and treated 10 AIDS patients with malignant lymphoma between January 2008 and December 2010. The diagnostic criteria used were from the diagnostic standard of adult HIV/AIDS formulated by the U.S. Centers for Disease Control and Prevention (CDC) in 1993. The diagnostic criteria for malignant lymphoma used were from the Tumor Section of the Chinese Medical Association Clinical Practice Guidelines. The antibody test for AIDS used confirmatory Western blot conducted in the lab of the local Center for Disease Control and Prevention to confirm the presence of positive HIV-1 antibodies.

### Immunoassay

The CD4^+^ cell count was determined in the research laboratory of the Beijing Ditan Hospital. FACS Calibur flow cytometry was used in the test; the instrument and reagent were supplied by the U.S. BD company. Reagents used in the test were TriTEST three-color reagent, MultiTEST four-color reagent, and FACS hemolysin. The quality was controlled by absolute counting. To test for malignant lymphoma, abnormal tissues from different locations, including lymph node, gastric mucosa, colonic mucosa, galactophore, and brain were analyzed through histopathology, cytology, and immunohistochemical detection. Clinical staging was based on the Ann Arbor clinical stages program.

### Statistical analysis

Student’s *t*-test or variance analysis was used for data analysis. SPPS 10.0 software was used.

## Results

### Epidemiology and clinical manifestation

One case was infected through blood, 3 cases were infected through sexual intercourse, and 3 cases had unknown routes of transmission. Five cases had enlargement of superficial lymph nodes, 2 cases were accompanied by fever, 2 cases had intermittent fever and 3 cases had abdominal pain and distension accompanied by fever.

### Clinical data and treatment outcome of patients

Please see **Table 1**.

**Table 1:** Clinical data and treatment outcome of patients.

No.	Gender	Age, years	Course of disease	Primary site	HAART	Chemotherapy	Survival time	Outcome
1	Male	46	3 months	Axillary nodes	AZT/3TC/NVP	VP/CODOXM/IVAC	>1 year	Partial remission
2	Female	40	1 month	Stomach	d4T/3TC/NVP	R-CHOP/R-MINE/MINE	1.5 years	Complete emission
3	Male	56	1 year	Adrenal glands	d4T/3TC/NVP	CHOP	<1 year	Died of renal failure
4	Male	57	2 months	Cervical lymph node	d4T/3TC/EFV	R-CHOP	No follow-up	Improved
5	Female	30	20 days	Breast	Non	Abandoned treatment	1 month	Died of lymphoma
6	Male	30	1 month	Cervical lymph node	d4T/3TC/EFV	Recommended CHOP+/-E	No follow-up	Home treatment
7	Male	28	1 month	Cervical lymph node	Non	Abandoned treatment	2 months	Died of lymphoma
8	Male	31	1 year	Encephalic	d4T/3TC/NVP	Dexamethasone	2 months	Died of lymphoma
9	Male	48	2 months	Axillary nodes	Non	CHOP	2 months	Died of toxic shock
10	Male	48	1 year	Colon	Non	CHOP	6 months	Died of severe anemia

d4T: stavudine; AZT: zidovudine; 3TC: lamivudine; NVP: nevirapine; EFV: stocrin. VP (vincristine + prednisone); CODOXM (cyclophosphamide + vincristine + pirarubicin+ cytarabine + methotrexate + calcium folinate); IVAC (cyclophosphamide + mesna + etoposide + cytarabine); CHOP (cyclophosphamide + adriamycin + vincristine + prednisone); R-CHOP (rituximab + cyclophosphamide + adriamycin + vincristine + prednisone); R-MINE (rituximab + ifosfamide + mitoxantrone + etoposide + prednisone); E: etoposide

### Laboratory examination

The confirmatory HIV antibody test for the 10 cases yielded positive results; whereas the CD4^+^ cell count of 8 patients were less than 200/µL. For 2 patients, the CD4^+^ cell count was more than 200/µL. Seven cases were negative for EBV-IgM, whereas 3 cases were not examined.

### Pathology results and clinical stage

**Figure 1:**
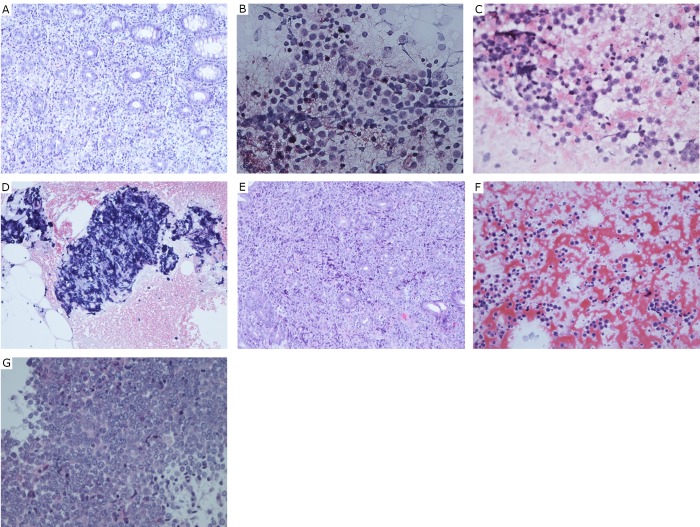
A: Case 10 (narrow intestinal lumen 35-40 cm from the anus). Four miliary colonic tissue samples were collected, one of which showed active chronic inflammation. A heterocyst can be seen in the other three pieces; B: Case 7 (punctate smear of cervical lymph nodes). A large quantity of dysplastic lymphoblasts indicated diffuse large B cell lymphoma. Immunohistochemistry: CD20(+), CD3(-), CD10(-), MUM-1(-), Bcl-6(-), Ki-67(-), Lambda(±), Kappa(±); C: Case 6 (punctate smear of lymph nodes). Cellular degeneration was obvious. Small quantities of dyskaryotic cells, and sometimes nuclear division figures were observed; D: Case 5 (galactophore puncture cell mass). Burkitt’s lymphoma. Immunohistochemistry: CD56(-),CK AE1/3(-), LCA(CD45)(+), CD3(-), CD20(+), CD10(+), Mum-1(-), Bcl-6(-), ki-67 (diffuse+); E: Case 2 (lower part of stomach), two miliary fundus glands from gastric mucosal tissues. Large quantities of atypical lymphocytes infiltrated into the mucosa, indicating non-Hodgkin’s diffuse large B cell lymphoma. Immunohistochemistry: tumor cell Bcl-6(+), CD10(+), CD20(+), CD3(-), CK AE1/3(-), Ki-67(+), Mum-1(+); F: Case 1 (left axillary lymph node). Needle aspiration cell mass: most active hyperplastic lymphocytes were active mother cells. Combined with immunohistochemistry, the lesion was diagnosed as Burkitt’s lymphoma. Immunohistochemistry: Bcl-6(+), CD10(+), CD20(+), CD3(-), Ki-67(+), Mum-1(-); G: Case 4. Fine-needle aspiration cytology: a single allotype macrolymphocyte indicated lymphoma. Combined with the immunohistochemistry result, the lesion was diagnosed as diffuse large B cell lymphoma. Immunohistochemistry: CK AE1/3(-), CD20(+++).

Please see **Table 2** and **Figure 1**.

**Table 2:** Patients’ pathology results and clinical stage.

No.	Location	Pathology results	Immunohistochemistry	Stage	Outcome
1	Axillary nodes	Burkitt’s lymphoma	BCL-6(+), CD10(+), CD20(+), CD3(-), Ki-67(+), Mum-1(-)	III ES	Remission after treatment
2	Stomach	Diffuse large B cell lymphoma	BCL-6(+), CD10(+), CD20(+), CD3(-), CKAE1/3(-), Ki-67(+), Mum-1(-)	IV	Remission after treatment
3	Adrenal gland	Diffuse large B cell lymphoma	CD20(+++), CD3(-), BCL-6(+-), CD10(-), Mum-1(-), CD5(-), Ki-67(+60%)	IV	Dead
4	Cervical lymph node	Diffuse large B cell lymphoma	CKAE1/3(-), CD20(+++)	IV	Improved, no follow-up
5	Breast	Burkitt’s lymphoma	CD56(-), CKAE1/3(-), LCA(CD45)(+), CD3(-), CD20(+), CD10(+), Mum-1(-) Bcl-6(-), Ki-67(diffuse+)	IV	Dead
6	Cervical lymph node	Burkitt’s lymphoma	CD10(+), CD20(+), EBER(+), Bcl-6(+), Ki-67(+)>95%	II E	Left the hospital
7	Cervical lymph node	Diffuse large B cell lymphoma	CD20(+), CD3(-), CD10(-), MUM-1(-), Bcl-6(-), Ki-67(-), λ(±), κ(±)	IV	Dead
8	Encephalic tissue	Diffuse large B cell lymphoma	Unavailable (IHC was done in another hospital)	IV	Dead
9	Axillary nodes and gastric mucosa	Diffuse large B cell lymphoma	Bcl-6(±), CD10(-), CD20(+), CD3(-), CD43(-), Ki-67(+), Mum-1(-)	IV	Dead
10	Colon	Lymphoma from B cell	CD20(+), Ki-67(+)	III	Dead

### Complications and untoward effects

Seven cases had pulmonary infections, 1 case had cryptococcal meningitis, 1 case had pyocyanic septicemia, 1 case had pulmonary tuberculosis, 2 cases had acute pancreatitis, 1 case had skin infection, and 3 cases had mycotic stomatitis. Moreover, 1 case developed central nervous system infection, 1 case had fungemia, 1 case had deep mycotic infection, 2 cases had hemorrhage of the digestive tract, 2 cases of renal dysfunction, and 4 cases developed bone marrow depression during chemotherapy. These complications or untoward effects often occurred simultaneously.

## Discussion

AIDS-related lymphomas (ARLs) occur mainly among more advanced AIDS patients. The CD4^+^ cell count in the peripheral blood is often lower than 100/µL. Therefore, the occurrence of lymphoma is related mainly to the degree and duration of the defect in the immune function of patients ^[^^3^^]^. Studies have shown that ARLs occur within 4 months to 27 months for AIDS patients ^[^^4^^]^. In this study, the CD4^+^ cell count in 8 patients was less than 200/µL. Further examination proved this finding.

Studies show that 95% of ARLs are from B cells ^[^^5^^]^, which are mostly highly malignant lymphomas, such as Burkitt’s lymphoma (BL), which accounts for 60%. The others are moderately malignant lymphomas, which mainly include diffuse large B cell lymphomas (DLBCLs) ^[^^6^^]^. Low-grade malignant lymphomas are rare. In the current study, 7 patients had DLBCL and 3 had BL. The data seems inconsistent because of the limited research data. Nevertheless, our results show that NHL develops from B cells.

ARL has several clinical manifestations. At the onset, B cell symptoms are common. At least 80% of patients are already in the stage IV at the onset of ARL, which is different from those among patients with the same type of lymphoma but without HIV infection. The latter is characterized mainly by painless enlargement of superficial lymph nodes. Only a small number of patients have B symptoms at onset during early clinical stages ^[^^7^^, ^^8^^]^. Seven patients had fever. Lymph nodes involvement occurred in 5 patients and extranodal involvement occurred in 5 patients, all of whom were in stage II E to stage IV. Lesions in the gastrointestinal tract, liver, and lung are the most common extranodal lesions of ARL. On the other hand, lesions rarely occur in the adrenal glands, breast, and the brain. In this report, invasion was observed in the gastrointestinal tract, the liver, the lungs, pancreas, the adrenal glands, breast, and the brain, which indicated that the patients were already in the middle or late stage of lymphoma.

The results of the EBV antibody examination were negative in 7 cases. Considering patients’ poor immune status, no further EBV-DNA examination was performed. For EBV-related lymphadenitis, whether the lymphoma was related to EBV infection could not be determined based on one case of lymph node pathology. The study showed that many ARLs were related to EBV infection and that the relationship between them was related to the type of lymphoma ^[^^9^^, ^^10^^]^. Data also showed that 79% of immunoblast or large cell lymphoma cases are positive for EBV and only 40% of Burkitt’s lymphomas are positive for EBV. In this study, 70% of the patients had DLBCL and only 30% were Burkitt’s lymphomas.

Before highly active antiretroviral treatment (HAART) was applied in clinical practice, chemotherapy in small dosages was always used for the treatment of ARL to avoid the toxicity of chemotherapeutic drugs. The 2-year survival rate was about 10% ^[^^9^^, ^^11^^]^. After HAART was applied widely in 1996, the immune function of patients could be restored, thus, ARL patients could endure chemotherapy at standard dosages without the amplification of untoward effects ^[^^12^^, ^^13^^]^. However, whether HAART should be used in chemotherapy synchronously is still under debate. Many scholars believe that the application of HAART in the process of chemotherapy is safe and effective ^[^^14^^, ^^15^^]^. In this study, 6 patients chose chemotherapy. Three cases underwent chemotherapy after HAART was applied, 1 underwent HAART and chemotherapy at the same time, and 2 underwent chemotherapy 1 or 2 times without HAART. Four patients did not undergo chemotherapy whereas 2 of them chose to be treated with HAART. Two of the 3 patients treated with HAART and subsequent chemotherapy are now in remission, whereas 1 died of a recrudescent tumor. The patient treated with HAART and chemotherapy at the same time left hospital without follow-up. One of the 2 patients who chose to be treated with HAART alone left the hospital without follow-up whereas the other patient died. The 2 patients who underwent chemotherapy only 1 to 2 times also died. The 2 patients who did not undergo any treatment died. These results indicate that although ARL patients have bad prognosis with a high death rate, HAART in combination with chemotherapy at the standard dosage produces better prognosis than simple chemotherapy. The studies about this aspect have more data to be accumulated for further deep investigation.

The complications which developed among patients during chemotherapy included bacterial and mycotic infections at different locations, tuberculosis, and bone marrow depression, hemorrhage of the digestive tract, and renal dysfunction. These complications indicate that chemotherapeutic drugs cause further injury to the immune function of AIDS patients, resulting in the occurrence of various opportunistic infections. The bone marrow depression caused by chemotherapeutic drugs is another reason for the multi-site infections. In addition, the toxicities of chemotherapeutic drugs to the liver and kidney injure important visceral organs and affect the prognosis of patients. Timely anti-infective and symptomatic treatments are the key factors for chemotherapy. However, the overall prognosis of patients is not very optimistic. In this report, 3 patients died of chemotherapeutic complications, whereas 3 died of lymphoma.

In summary, AIDS related lymphoma, a common malignant tumor, always occurs among advanced AIDS patients. The most dominant type of lymphoma is NHL from B cells. Although AIDS related lymphoma presents various types of clinical manifestations, it is often indicated by invasion of visceral organs. Patients are always in the middle and late stages of lymphoma when they consult a doctor, making effective treatment difficult. Hence, the overall prognosis of patients remains bad even when HAART treatment is used. We emphasize awareness of related diseases, as well as early detection and treatment to improve the patient prognosis.
